# In Vitro Veritas: From 2D Cultures to Organ-on-a-Chip Models to Study Immunogenic Cell Death in the Tumor Microenvironment

**DOI:** 10.3390/cells11223705

**Published:** 2022-11-21

**Authors:** Dmitri V. Krysko, Robin Demuynck, Iuliia Efimova, Faye Naessens, Olga Krysko, Elena Catanzaro

**Affiliations:** 1 Cell Death Investigation and Therapy Laboratory, Department of Human Structure and Repair, Ghent University, 9000 Ghent, Belgium; 2 Cancer Research Institute Ghent, 9000 Ghent, Belgium; 3 Institute of Biology and Biomedicine, National Research Lobachevsky State University of Nizhny Novgorod, Nizhny Novgorod 603022, Russia

**Keywords:** immunogenic cell death, tumor microenvironment, 3D cell culture, spheroids, organoids, bioperfusion bioreactors, organ-on-a-chip

## Abstract

Immunogenic cell death (ICD) is a functionally unique form of cell death that promotes a T-cell-dependent anti-tumor immune response specific to antigens originating from dying cancer cells. Many anticancer agents and strategies induce ICD, but despite their robust effects in vitro and in vivo on mice, translation into the clinic remains challenging. A major hindrance in antitumor research is the poor predictive ability of classic 2D in vitro models, which do not consider tumor biological complexity, such as the contribution of the tumor microenvironment (TME), which plays a crucial role in immunosuppression and cancer evasion. In this review, we describe different tumor models, from 2D cultures to organ-on-a-chip technology, as well as spheroids and perfusion bioreactors, all of which mimic the different degrees of the TME complexity. Next, we discuss how 3D cell cultures can be applied to study ICD and how to increase the translational potential of the ICD inducers. Finally, novel research directions are provided regarding ICD in the 3D cellular context which may lead to novel immunotherapies for cancer.

## 1. Introduction

**Figure 1:**
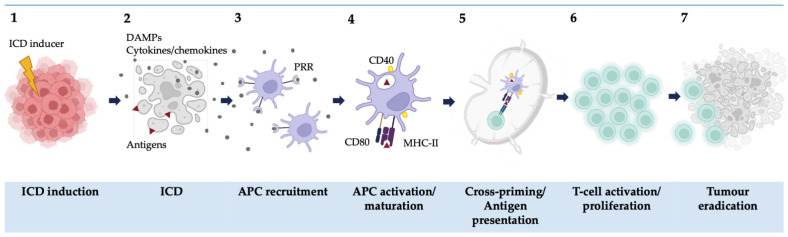
Immunogenic cell death (ICD): an overview. ICD can be induced in cancer cells (including by chemotherapeutics, radiation, photodynamic therapy) (**1**) and it is characterized by the expression of tumor-specific antigens (antigenicity) and the spatiotemporal emission of DAMPs, cytokines, or chemokines (adjuvanticity) (**2**). The combination of this antigenicity and adjuvanticity allows the recruitment of antigen-presenting cells (APCs) via their pattern recognition receptors (e.g., TLR4) (**3**). The APCs undergo activation and maturation which is associated with the expression of CD40, CD80, and MHC-II (**4**). After migrating to the local lymph nodes, the APCs present the antigens to the T-lymphocytes (**5**). The activated T-cells, mainly CD8 cytotoxic T-cells, proliferate (**6**) and elicit a tumor-specific immune response by migrating to the tumor site, causing tumor eradication (**7**) and T-cell memory formation.

The immunogenicity of dying neoplastic cells has been clearly recognized as a critical determinant of the efficacy of cancer therapy [1,2,3]. In 2005, Casares et al. [4], for the first time, introduced the concept of immunogenic cell death (ICD) (Figure 1). This is an umbrella term covering different regulated cell death modalities, such as apoptosis [5,6], ferroptosis [7,8], and necroptosis [9,10]. These processes kill cancer cells and restore the lost immunological ability to identify and interact with cancer cells [2,3,5,11] (Figure 1), but their molecular pathways have not been fully identified. Thus, only some apoptotic, ferroptotic, and necroptotic inducers stimulate the innate and adaptive immune responses, leading to the establishment of long-term anti-cancer immunological memory [5].

Since 2005, it has been demonstrated that ICD results from a correct balance between the recognition of antigenic structures (antigenicity) and the spatiotemporal emission of immune-modulatory mediators (adjuvanticity) [1,12]. Adjuvanticity is mediated by the emission of damage-associated molecular patterns (DAMPs) and proinflammatory cytokines/chemokines from the dying cancer cells, which stimulate corresponding pattern recognition receptors (PRRs) of the innate immune system. DAMPs are normally retained within cells and participate in their normal functioning [2]. However, once released outside the cells, they act as danger signals [2,13] (Figure 1). The secretion/release of DAMPs must follow strict spatiotemporal kinetics and is triggered by concerted PERK-EIF2α-mediated endoplasmic reticulum (ER) and oxidative stress [6,14,15].

Some anticancer agents routinely used in the clinic have been found to be ICD inducers (e.g., doxorubicin and mitoxantrone) but not all of them always act as anticancer vaccines. Furthermore, new inducers that trigger ICD in vitro and in vivo have failed in clinical trials. Many explanations have been offered to explain this lack of response, which has impeded their translation into the clinics. The most accepted explanation is that the timing and drug dosage schedule are crucial in promoting the activation of an adaptive immune response [16]. This is not surprising because ICD is a highly tuned process and, for instance, early but not late ferroptotic cancer cells can induce ICD [7,17].

Besides the therapeutic schedule, the TME also can play a crucial role in immunosuppression and/or immune activation and thereby affect the therapeutic outcome. The TME is a combination of molecules, cells, tissues, and structures (e.g., blood vessels) that surround the actual tumor. The TME compositions differ among tumors type, but it always includes stromal and immune cells, blood vessels, and the extracellular matrix (ECM). Altogether, the TME components constantly communicate with the tumor and influence its growth and spread. Indeed, the role of TME in tumorigenesis and therapeutic responses is crucial. Depending on the TME composition, it can have anti- or pro-tumorigenic properties. For instance, the quality of infiltrated immune cells will decide the fate of immunological status, i.e., if it is either immunosuppressive or immunostimulant. Stromal cells, such as fibroblasts, endothelial cells, or adipocytes, are responsible for activating signaling pathways involved in cancer cell sustaining and propagation, such as angiogenesis, proliferation, invasion, and metastasis. Fibroblasts, for example, represent one of the most abundant cell populations found in the TME, which contribute to the formation of the connective tissue structure and function through the secretion of important ECM components such as collagen, fibronectin, laminins, glycosaminoglycans, and proteoglycans. ECM, in its turn, has a multifaced role which does not exhaust in silent structural support. On the contrary, it activates biochemical pathways crucial for the regulation of the cell–cell and cell–matrix crosstalks and results in the control of tissue morphogenesis, differentiation, and homeostasis [18,19].

Despite the well-defined importance of the TME in response to anti-tumor immunotherapy, not many preclinical studies take into consideration the mutual impact of the TME and ICD in the cancer immunization process. Thus, it is not known if and how ICD can modulate the TME or vice versa. Nevertheless, cancer cells reside in a complex environment with a heterogeneous set of stromal cells and depend on close interactions with various components of the TME. This repertoire of cells that are recruited differs among tumor types and contributes to the acquisition of the hallmarks of cancer [20]. Early in tumor growth, tumor cells trigger significant molecular, cellular, and physical changes, creating a dynamic and reciprocal relationship between cancer cells and components of the TME; this relationship supports cancer cell survival, local invasion, and metastatic dissemination [18]. It is accepted that the TME is not simply a silent bystander but an active promotor of cancer progression [18]. In contrast to the once-dominant tumor-centric view of cancer, the concept of the involvement of the TME has proven to be extremely useful because it has contributed to identifying and understanding the role of non-genetic and non-cell-intrinsic factors in cancer development [18]. However, the main caveat of perpetuating this model is the lack of a comprehensive characterization of the ICD–TME interaction and the absence of a comprehensive in vitro model that recreates the TME. Thus, the aim of this review is to describe and discuss different in vitro methods (from 2D to organ on-a-chip) that might facilitate the translation of ICD-inducing drugs to the clinic. Many reviews have shown the pros and cons of the in vitro TME models most widely used to study immunotherapy in general [21,22]. For this reason, in this review, we will only describe the models that are more relevant for ICD studies and discuss how they can be exploited in future research.

## 2. ICD in 2D Models

The vast majority of in vitro studies are carried out on 2D models. However, flat cultures do not consider the whole tumor complexity represented by the TME, and studying ICD and TME in this context is quite difficult. The TME is a complex multicellular setting in which all components of the TME interact with each other. Recreating such an environment in vitro must take into account cellular heterogeneity (immune cells, fibroblasts, etc.), signaling, and communication within the tumor. Therefore, it is not feasible to generate this highly organized setting in flat and simple 2D models.

**Figure 2:**
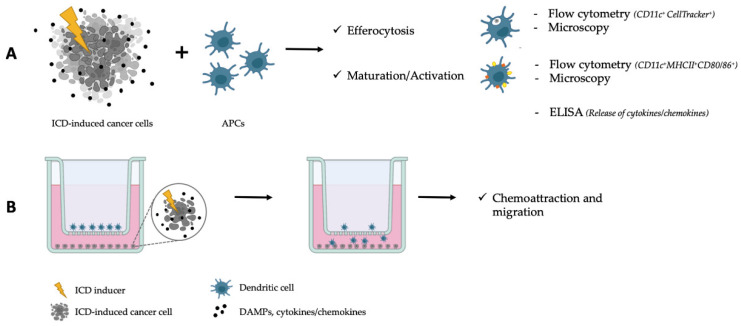
Two-dimensional models to study ICD. (**A**) Co-cultures of cancer cells ongoing ICD and antigen presenting cells (APCs) can be used to study efferocytosis and the APC status (e.g., maturation/activation). (**B**) Chemoattraction and migration can be assessed through Transwell migration assays.

One of the few models currently used to reflect the TME in the context of ICD is the use of mixed co-cultures of cancer cells and antigen presenting cells (APCs) to study the specific interactions between cancer and the immune system (i.e., phagocytosis [efferocytosis] [23], maturation/activation of APCs, etc.), allowing the immune system to detect the dying/dead cancer cells and respond to them or ignore them (Figure 2). The efferocytosis of dying/dead cancer cells by professional or non-professional phagocytes can be assessed [24,25] (Table 1). Importantly, there are different cell types in the TME, all of which can participate in the efferocytosis of dying cancer cells. Two-dimensional efferocytosis assays can be performed either with cell lines (Raw 264.7 macrophages [26], THP-1 monocytes [27], etc.) and/or with primary APCs (bone-marrow derived macrophages (BMDMs [26]) or bone-marrow derived dendritic cells (BMDDCs), BMD monocytes [28], and peritoneal macrophages) which are co-cultured with target dying cancer cells [7,26].

Another way to study the interactions of APCs and T cells with dying cancer cells in a 2D system is indirect co-culture using Transwell systems (Figure 2). In these systems, T cells [29] and cancer cells [30] (or their supernatants) are seeded and cultivated in the bottom chamber of plates, and APCs [30,31] (or TAMs [29]) are seeded in Transwell inserts, which have microporous membranes, allowing media exchange and cell communication between the plate well and the insert. If the APCs or TAMs migrate through the Transwell insert pores, the migration flux can be measured [29,31,32], hence monitoring the interaction and attraction of cells towards each other. Often, this migration of APCs and TAMs is stimulated by the chemotactic molecules emitted by dying cancer cells [33].

Besides direct or indirect cell-to-cell interactions and communications, the TME is characterized by altered cellular metabolism, i.e., hypoxia, acidosis, glucose, amino and fatty acids, lipids, etc. These factors can have an acute or a chronic effect, which will influence the response of the cells to every stimulus, including cell death [34,35]. It is noted that 2D models allow the recreation of many of these conditions. Hypoxia is frequently established by using particular types of chambers and by limiting the O_2_% to ≤ 5 (0–2% is most common for the TME) [36,37] by culturing the cells in a gas chamber with an oxygen sensor and using N_2_ to deplete oxygen [38,39,40,41]. When introducing acidosis, the cells are cultured in media with pH < 7.4 (6.4–6.8 pH is the average for TME experiments) [34,42,43,44,45]. Glucose deprivation in 2D cancer cells is performed by culturing the cells in glucose-free [46,47] or low-glucose [35] media (Table 1).

All these 2D models can take into consideration only a few parameters and are far from being representative of the TME. Therefore, to better integrate the complexity of the TME in the models, 3D structures need to be considered.

## 3. 3D Models

It is now widely acknowledged that 3D cultures are more representative of the in vivo microenvironment than 2D models [48]. By using hetero-cellular 3D cultures, the heterogenous character of the in vivo TME is clearly reflected. Intercellular contacts are recreated, the realistic expression of molecules is achieved (e.g., adhesion molecules, cytokines, growth factors, etc.), and hypoxic cores and diffusion gradients are formed, all of which have a considerable impact on the tumor behavior and therapeutic response [49]. For instance, the presence of intercellular contacts strongly affects the response of cancer cells to cell death stimuli; the response rate is much lower if the cells are seeded in 3D models [45,50]. Furthermore, some adhesion molecules are upregulated in tumors, and 3D models reflect their actual expression in the TME. E-cadherin (E-Cad) is one of the most important adhesion molecules, as it might cause chemotherapy resistance in different cancer types, such as ovarian cancer [51]. In contrast to ovarian cancer flat cultures, E-cadherin was overexpressed in ovarian cancer spheroids, which made it possible to study its impact on tumor cell behavior. E-cadherin overexpression might have been caused by tighter cell–cell contacts, forming a denser barrier to the drugs, making it more difficult for the drugs to reach all the cells in the tumor. Moreover, CD44 was found to be upregulated in spheroids [52]. This marker is associated not only with cell–cell interaction, but also with increased chemotherapy resistance, as in the case in real tumors [53]. Taking all these notions together, 3D cultures can be more readily correlated to patients and use 3D models in preclinical research might speed up the translational time of ICD research to clinical studies.

In the following sections, the different methods to model the TME in 3D will be discussed, following an increasing complexity order.

### 3.1. Spheroids

Spheroids are widely used to model tumor-like structures [48,54]. They are defined as spherical multicellular aggregates growing in suspension and containing a gradient of nutrients and oxygen, thus allowing them to reliably mimic in vivo tissue [54]. Different methods for building spheroids include, but are not limited to, ultra-low attachment plates, hanging drop methods, microfluidics, and spinner cultures [48,54,55]. In ultra-low attachment plates, cells are seeded as a single-cell suspension, and as they cannot adhere to the plate, they form spheroids [56]. This method allows high-throughput spheroid formation, but it cannot control spheroid size. To solve this, agarose molds with defined dimensions can be used to enable the high-throughput generation of uniform spheroids [48]. On the other hand, this agarose mold method cannot be used to form bigger spheroids because the spheroids cannot grow out of the constraints of the pre-defined mold. The ultra-low attachment plate and the agarose mold methods are both cheap, fast, and easy to use in order to simultaneously generate many spheroids. The hanging drop method is more technically challenging and cannot be used for high-throughput spheroid formation because only one spheroid per drop can be formed [57]. The use of microfluidics is another way to generate spheroids, but it is expensive and technically challenging. It can be used for the high-throughput generation of uniform spheroids, but many parameters need to be optimized for stable spheroid formation [58]. Spinner cultures utilize centrifugal forces to keep cells in suspension and “push” them together to allow spheroid formation [59]. This method is not often used anymore and cannot generate uniform spheroids.

In general, the main principle in the generation of spheroids is based on self-assembly and self-organization. Regardless of the method used to build the spheroids, they can be characterized by several levels of complexity, depending on whether they contain one cell type (monocellular) or more than one cell type (heterocellular) [60]. Monocellular spheroids represent the simplest 3D model. Cells are cultured in non-adhesive surroundings to allow the cells to assemble in a compact spheroid. It should be noted that not all cell types are suitable for spheroid generation and not all methods can generate compact spheroids [61]. Depending on the spheroid size, its core might be hypoxic to a certain degree due to the absence of oxygen and decreased nutrient supply, resulting in a necrotic core, as observed in in vivo tumors [48]. Due to the increased cell contacts in spheroids, the cells change their phenotype, metabolism, and responses to cell death [48,62]. This includes, but is not limited to, an increase in ECM production. For instance, in carcinoma spheroids generated by seeding cells in non-adhesive 96-well plates, the expression levels of lumican, SNED1, and DARP32, which are all well-known matrix proteins, were increased when compared to standard 2D models of the same cancer type [56]. Cells also shifted to an increased glycolytic metabolism in 3D and had decreased drug sensitivity to most drugs. Indeed, spheroids are much less responsive to cell death inducers when compared to 2D models [48], making it a better model for studying the in vivo therapeutic response. In addition, when comparing cell death responses in 2D cultures of MCA205 fibrosarcoma cells and SKOV ovarian cancer cells with spheroids, it was found that resistance to (ferroptotic) cell death increased over time during spheroid formation [48]. This clearly shows how 2D cultures do not reflect the therapy responses in patients; spheroids are more reliable for mimicking drug responses. However, tumors consist of different cell types that interact with each other, and this heterogenicity inevitably alters the final response to therapy. Therefore, other models were built based on monocellular spheroids to make this model more representative of the in vivo TME.

One way to improve the relevance of spheroids is to combine two or more different cell types to generate heterocellular models. By using different cell types together with cancerous cells, a more realistic tumor model is obtained. Of course, a tumor consists of more than one cell type (e.g., cancer-associated fibroblasts (CAFs), macrophages, etc., are present), and cells inside the TME can be polarized towards a pro-tumor phenotype [20]. In addition, heterocellular 3D structures can self-assemble and self-organize into different structures. For example, when combining fibroblasts with endothelial cells (HUVEC heterogenous spheroids allow the formation of vascular networks) [60], vascular networks are formed and lumen formation was observed in more mature spheroids (on day 10 after spheroid formation). In a more relevant setting, pancreatic cancer cells were combined with CAFs in ultra-low attachment plates to form heterocellular spheroids [63]. These spheroids were treated with a combination of radiotherapy and photodynamic therapy to assess tumor growth and necrosis after treatment. This study showed that the combined treatment reduced tumor growth while simultaneously increasing tumor necrosis. This phenomenon would not have been observed in simple 2D models, which emphasizes the relevance of spheroid models. Even more than two cell types can be combined in order to include immune cells in the tumor model. For example, a spheroid consisting of non-small-cell lung cancers, CAFs and monocytes was constructed [64]. The tumor spheroid was generated by using spinning culture and mixed with a single-cell culture of CAFs and monocytes, and subsequently encapsulated in alginate (see next section). In this model, the monocytes infiltrate the tumor and polarize towards an M2-immunosuppressive phenotype, recapitulating their polarization in vivo. These data together indeed show that 3D models can closely mimic the in vivo TME by recapitulating the immunosuppressive phenotype interfering with therapy efficacy.

### 3.2. Complex 3D Models Which Include ECM

One of the limiting factors of spheroids is the scarce ECM secretion [65]. Therefore, new tools have been developed to mimic the in vivo ECM, mainly the usage of biomaterials (see Box 1). The ECM is an important factor in the TME as this provides an extra physical barrier to drugs and thus limits their efficacy. It can also communicate with cells via different receptors (e.g., integrin receptors). It is known that increased signaling via the integrin pathway can induce proliferation and cell survival by inhibiting cell death [66]. Besides cells binding to the ECM, certain drugs can also bind molecules present in the ECM. For instance, it was found that cisplatin, an often-used chemotherapeutic, extensively binds collagen, a major constituent of the ECM, which limits its diffusion into cancerous cells [67]. The tumor ECM also provides certain cues, such as increased matrix stiffness, which drive quiescence and make the cells more resistant to cell death [68]. As the tumor ECM is stiffer due to increased secretion of collagen, fibronectin, and other proteins, this should also be reflected in the 3D tumor model to make it more representative and useful [69]. By using different biomaterials, the stiffness of the ECM can be adapted to obtain a microenvironment resembling the in vivo TME [68]. In breast cancer, for example, stiff matrices together with hypoxia modulate stemness [70]. It is known that cancer stem cells are more resistant to drugs and can cause relapse [71]. As mentioned above, ECM secretion is enhanced in spheroids (compared to 2D models), which partially limits the diffusion of drugs into the spheroid and limits their cell death responses [48]. However, this is rather limited, and exploiting biomaterials in 3D models can more effectively mimic the ECM, and thus the TME [56,65].

Box 1 **Most common biomaterials used to recreate the TME.**

Understanding the composition and function of the ECM is not only crucial to obtain tumor basic knowledge but also for the development of 3D models. Matrix and matrix scaffolds enable the study of microenvironmental cues including cell–cell and cell–matrix interactions. Different biomaterials with different properties can be used to create several variants of specific TMEs. However, what they need to have in common is to be biocompatible with the cells to allow those cells to interact with the ECM.

Here are some examples of biomaterials:

**Hydrogels**, such as gelatin methacrylamide (GelMOD or GelMA), represent a hydrophilic polymer network [72], which are highly biocompatible and allow cellular infiltration, making them important for tumor progression. Hydrogels can be natural (e.g., collagen) or synthetic (e.g., poly-ethylene glycol) and reversible or permanent. Natural hydrogels offer excellent biocompatibility and biodegradability, but they often lack sufficient mechanical stability. In contrast, synthetic polymers have higher mechanical strength but lack biocompatible properties. Furthermore, reversible hydrogels have weaker chain–chain interactions (i.e., the biomaterial reverts from the solid gel-like state to a solution) by changing certain conditions such as pH or temperature, whereas the crosslinking in permanent hydrogels cannot be undone (Table 2).

A natural and reversible biomaterial that is often used is **collagen**. This class of fibrous proteins is often used in cancer research to mimic the in vivo ECM [73], which has led to the finding that the density of the collagen matrix influences the proliferation of T-cells [74]. This indicates that tumors induce immune suppression, not only by releasing immunosuppressive factors but also by remodeling the ECM to alter T-cell responses.

**GelMOD** is a semi-synthetic biopolymer that can polymerize after irradiation with UV light in the presence of a photo-initiator. Gelatin is derived by the (partial) hydrolysis of collagen [75], and the methacrylamide functional groups introduced into the gelatin backbone make it possible to crosslink the gelatin strands. As an example, GelMOD has been used to model the blood–brain barrier during brain metastasis [76].

In addition, **Matrigel**, a biomaterial derived from the ECM of mouse sarcoma, which mimics the mammalian basal cell matrix, can be used for tumor modeling [77,78]. The application of Matrigel in cancer research has already led to many discoveries; however, it is essential to choose a representative biomaterial because this choice could heavily impact the results [79]. Cancer cells usually secrete matrix metalloproteases to overcome barriers presented by the ECM. Yet, it was discovered that when Matrigel is used, cancer cells do not need metalloproteases to overcome the barrier, whereas with a collagen matrix, metalloproteases are essential [80].

Biomaterials can also be combined to create a more complex environment that might better resemble the in vivo TME. In line with this, it has been found that **adding Matrigel to a collagen matrix** increases its stiffness, which leads to increased focal adhesions by cancerous cells [81]. This model might be more relevant for studying invasion and metastasis compared to Matrigel alone. A combination of **Matrigel and Alginate** has been used as well [82], which led to new in vitro breast cancer model construction. Alginate is a natural irreversible biomaterial that helps to stabilize the 3D structure over a long period of time, whereas Matrigel reflects the tumor ECM better and allows the in vivo cellular behavior to be recapitulated. In another study, a synthetic poly-lactic acid scaffold was combined with gelMOD and loaded with a co-culture of CAFs and ovarian or colon cancer cells in a collagen suspension [83]. This model was found to be an excellent tool for studying peritoneal metastases.

**Table 2:** The categorization, advantages (+), and disadvantages (−) of the most used biomaterials in bioengineering.

Biomaterial	Synthetic/Natural	(Non-)Reversible	(Dis)Advantages	References
Collagen	Natural	Reversible	+High density, natural −Batch-to-batch variation	[74,75]
GelMOD	Semi-synthetic	Reversible	+Biocompatible −Heterogenous network, shrinking during crosslinking	[77,84]
Matrigel	Natural	Non-reversible	+Mimics natural tumor ECM −Batch-to-batch variation, weaker material	[77,80]
Alginate	Natural	Non-reversible	+Stabilizes 3D structures −Difficult to work with	[82]
Poly-lactic acid	Synthetic	Reversible	+Strong material −Not natural	[83]

### 3.2.1. Organoids

Organoids are 3D structures organized in a specific spatial pattern to resemble miniature organs ex vivo. They can be engineered using biomaterial scaffolds or 3D bioprinting.

Distinguishing between organoids and spheroids is not always easy. Generally, one speaks of organoids when an organ-like architecture is present, e.g., in gut organoids. They are usually grown from stem cells and are frequently more representative of the TME [85]. Patient-derived cells can also be used to generate organoids; for instance, ex vivo samples can be considered organoids [86]. Tumor organoids can be formed by cultivating biopsies. This makes tumor organoids, often called tumorspheres, very promising models for investigating immune responses and therapy efficacy as they very closely resemble the in vivo tumor microenvironment. To grow organoids, stem cells are grown on a basal lamina consisting of biomaterials in order to allow growth, differentiation, and maturation [53]. Growing organoids is complicated, and they take a while to mature, making their analysis a complex and time-consuming process. However, they are more representative of the in vivo microenvironment, and since they can often be patient-derived, they may facilitate the progress of pre-clinical research towards the clinic.

In addition, since organoids are a better representation of tumor-like structures than 2D cultures and allow prolonged cell proliferation, they can be stored in biobanks. Indeed, not only is the architecture of cancer organoids similar to tumors, but they are also genetically similar. For example, a biobank composed of 17 normal and 46 gastric cancer organoids showed that the organoids resembled the original tumor on multiple levels, including genomics and transcriptomics [87]. This biobank also led to the discovery of multiple potential targets for therapy. Normal organoids were constructed by resuspending tissue pieces in Matrigel. The tissue–Matrigel construct was cultured in a medium containing several growth factors (Wnt3a, Noggin, EGF, etc.), and organoids started to form after approximately one week. Several optimizations were required to establish tumor organoids, e.g., by digesting the tissue and adding other factors to the growth media (e.g., Nutlin3a for tumors carrying TP53 mutations [88]). This need of organoids for multiple factors in order to form and proliferate shows the complexity of culturing them. However, the results obtained from organoids can be directly correlated to patients, which promotes progress towards clinical applications. Therefore, generating biobanks of different tumor organoids might facilitate the choice of effective treatments for cancer patients.

Besides the complexity of establishing organoid cultures, their success rate is low. For instance, in one study in which bladder cancer organoids were generated, only 50–80% of the cell lines formed organoids, and not every organoid that formed grew after passaging [89]. For hepatocellular carcinoma organoids, the success rate was only 26% [90]. The success rate of organoid culture is strongly dependent on the cancer type [84]. Colon, breast, lung, and liver cancer organoids have high success rates of 90%, 80%, 70%, and 100%, respectively [91], whereas bladder cancer and hepatocellular carcinoma organoids (previously mentioned) have low success rates [89,90]. Therefore, the generation of organoid cultures might not be feasible for every patient and every cancer type, which further complicates the potential clinical application of organoids. However, protocols have been established for the analysis of T-cell responses to tumor organoids, and hopefully in vivo tumors [92]. In that work, organoids were co-cultured with peripheral blood lymphocytes, and T-cells were expanded and evaluated for their tumor-killing capacity.

In another study that used the same co-culture setting, two patient-derived lung cancer organoids were exploited to confirm the ability of mitoxantrone and methylene blue photodynamic therapy to trigger ICD, which was first analyzed on 2D flat cells. In this elegant study, the abscopal effect of ICD was monitored by analyzing the involvement of CD8^+^ T cell reactivity and cytolysis via granzyme B in such organoids [93], demonstrating the high potential of this model.

Nonetheless, a limiting factor of organoids is the time to obtain them and the variable success rate between different cancer types. Organoids represent the next step in cancer research and more specifically in the ICD field, but more research is warranted into organoid establishment, such as the requirement of specific growth factors for the growth and survival of the many different cell types inside the organoid TME.

### 3.2.2. Perfusion Models

Despite the undeniable advantages of 3D cultures over traditional flat models, 3D cultivations still have some intrinsic limitations that do not allow the spatiotemporal distribution of oxygen, nutrients, and metabolic wastes to be mirrored and do not match the environmental conditions normally present in vivo. Consequently, cellular behavior would not be realistic [94]. For instance, the amount and distribution of soluble factors in vivo is characterized by spatial gradients that play an important role during proliferation, differentiation, and tissue development and cannot be recreated in 2D or static 3D models. Moreover, the exchange of cytokines and growth factors or immune cell infiltration represents the foundation of immune response communication, and static models cannot recreate it. All these limitations can be resolved using dynamic cell culture devices, i.e., perfusion bioreactors and organ-on-a-chip.

#### Perfusion Bioreactors

A perfusion bioreactor is a device that provides a highly controlled and reproducible tumor environment. The core of these structures is represented by an inlet and an outlet pipe for the fluid and a flow chamber for the scaffold that acts as a bridge between the two tubes. It consists of a glass vessel and scaffolds in which cell growth takes place, a set of sensors for measuring chemical and physical parameters (e.g., temperature, pH, oxygenation), peristaltic pumps for the automatic injection of appropriate solutions (acidic, basic, or nutritive), bottles as reservoirs of solutions to be introduced into the culture broth, and a control unit for monitoring and controlling the main parameters. By ensuring mechanical stability, scaffolds allow the production of an extra-cellular matrix that influences the physiological regulation of cellular function. This instrument ensures the nutrient exchange and removal of waste substances, as well as the application of appropriate physical and chemical stimuli in a measurable and controllable way to efficiently promote a spatial distribution of cells in 3D. The application of perfusion allows the active diffusion of nutrients and waste substances, ensuring physiological concentrations. Together with the real-time monitoring of dynamic cell–cell interactions, the efficient exchange of cytokines and growth factors makes this model effective in mirroring the in vivo environment. Perfusion bioreactors make it possible to enhance the proliferation, differentiation, and formation of new ECM, while also prolonging cell culture time without perturbations by external factors. Furthermore, many studies have shown that the cultivation of patient-derived biopsies in perfusion bioreactors can maintain the same gene expression profile and drug resistance patterns recorded in vivo [95]. Models of colon cancer [96,97], breast carcinoma [95,98,99,100], ovarian cancer [101], bone metastatic prostate cancer [102], Erwin sarcoma [103,104], leukemia [105,106], and glioblastoma [107] have been engineered using perfusion bioreactors.

The most interesting use for bioreactors is not the creation of ex novo replicas of TME, but the maintenance and culturing of primary cancer biopsies that preserve the TME. Perfusion bioreactors enable the cultivation of ex vivo samples for up to three weeks and capture the interaction between tumor cells, the extracellular matrix, and the immune system [95,106]. The perfusion flow pushes the culture media through porous scaffolds, providing more nutrients and oxygen to the tissue, which increases the survival of all cells in the TME, including tumor cells, lymphocytes, and stromal cells [95].

Some tumors are characterized by a suppressive TME more than others, which makes them resistant to immunotherapy. Thus, models that take into consideration both tumor cells and the surrounding populations make it possible to study the immunosuppressant TME and assess the effect of immunotherapy.

Using a U-CUP bioreactor and collagen scaffolds, it is possible to maintain breast cancer tissue biopsies. Muraro et al. [95] demonstrated that such preserved tumor cells do not carry any key alterations in cancer-driving genes, including estrogen receptor status, while maintaining the tumor’s heterogeneity. Tumor cells continue proliferating together with the tumor-infiltrating lymphocytes and other immune cells, including CD68^+^ macrophages, and CD3^+^ T and CD20^+^ B lymphocytes. This represents a very effective model to assess the different immunogenic potential of ICD inducers.

The same U-CUP can be used to maintain glioblastoma explants, assess the TME and tumor composition, and test the effect of checkpoint inhibitors. Brain tissue fragments were inserted in discs of microfibrillar collagen hemostat sheet, which were placed in silicone adaptors and ethylene–tetrafluoroethylene copolymer mesh grids and placed in the bioreactor. There were then perfused with a mix of cell culture media and growth factors. In this model, it was shown that a subset of cells was prone to the emission of IFNγ and consequently to shifts in immune cell composition within specified tissue portions; immune responder and non-responder subpopulations could then be identified [107].

A polydimethylsiloxane (PDMS) bioreactor was used to monitor the growth and therapeutic response of ovarian cell lines (SKOV-3, OVCAR-8, or CS-99) and patient-derived tumor samples. Cell lines and primary tumor cells were mixed into an ECM containing 90% bovine collagen type I and 10% basement membrane (matrigel with reduced content of growth factor) and cultivated in a perfusion biosystem. Interestingly, in this perfusion model, the cell lines cultivated with CAFs and/or peripheral blood mononuclear cells (PBMC), but not alone or in a static model, showed a similar cell proliferation rate and morphology to patient tissue and responded to chemotherapy. In addition, in the same perfusion bioreactor system, the patient-derived tumors maintained the same immune cells landscape for at least seven days, whereas chemotherapy increased cytotoxic and regulatory T cells [101]. Altogether, these results highlight the robustness of this platform for studying tailored co-cultures as a reliable preclinical model to test immunotherapy options.

Perfusion bioreactors also enable the creation of tumor niches in a 3D scaffold for prolonged ex vivo maintenance, such as bone marrow niches that support and drive leukemogenesis. Different studies reported the possibility of maintaining, expanding, and regulating human malignant hematopoietic stem and progenitor cells by culturing mesenchymal stromal cells derived from healthy human cord blood in porous scaffolds under perfusion [106,108]. This model can be used to recreate the spatial organization of hematopoietic stem and progenitor cells (attached to the niches) and mature cells (released into the circulation). To do that, the bioreactor can be loaded with myeloproliferative neoplasm CD34^+^ AML leukemia cells isolated from patients together with a stromal vascular niche by adding human stromal vascular fraction cells derived from adipose tissue [106]. In general, to investigate immunotherapeutic strategies or agents, greater complexity of the model results in a more reliable response.

Altogether, these results demonstrate the high potential of these models, especially keeping in mind their potential use for personalized targeted therapy. These models support xenograft growth and preserve the original immunophenotype, including the possibility of incorporating the original TME populations. Consequently, testing immunotherapy in perfusion bioreactors will help to deal with one of the major setbacks of immunotherapy, which is the individual lack of responsiveness due to the suppressive TME. For instance, biopsies from patients could be used to predict the efficacy of the therapy. However, tuning up perfusion bioreactor tumor models requires specific expertise, since many factors and parameters must be taken into consideration, such as the perfusion bioreactor model, the culture chamber, the scaffold type, and perfusion parameters. For instance, the culture chamber must permit the housing and maintenance of cells within a sterile environment. For this reason, it is crucial that the chamber components are easily autoclavable and, if possible, made from a transparent material so that the cells can be monitored. In addition, the chambers have to be easily accessible in order to seed the scaffolds with cells [109]. As for bio-printing, the choice of biomaterials for the scaffold is of much importance; they must not elicit adverse reactions in the tissues being cultured. To mimic the physiological microenvironment and the intrinsic characteristics of the extracellular matrix, the 3D scaffold must possess specific properties (such as porosity, biodegradability, and biocompatibility) and allow the bidirectional transport of nutrients, metabolic waste products, and oxygen. On top of that, the perfusion flow rate and the tumor load capacity must be optimized.

#### Tumor-on-a-Chip

**Figure 3:**
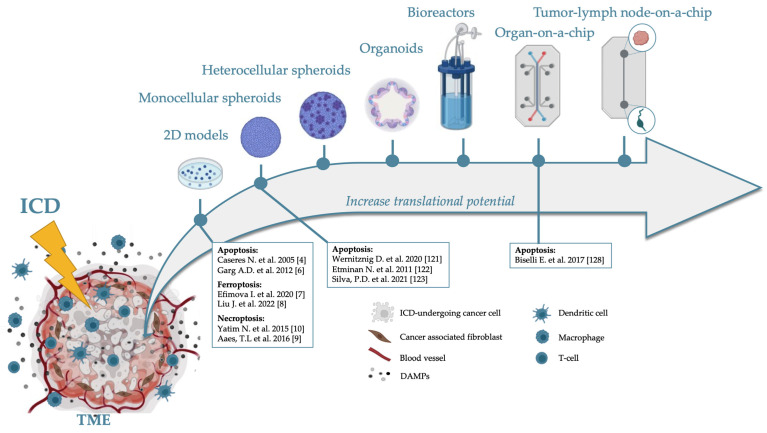
Available and implementable tumor models and devices used to study ICD within the TME.

One of the biggest limitations of organoids as models for studying anticancer immunotherapy is the lack of tissue-resident immune cells. One way to overcome this problem (besides using a perfusion bioreactor), and to introduce the possibility of including a vasculature system and a stromal component, is the tumor-on-a-chip technology. A tumor-on-a-chip consists of small amounts of tumor cell lines-derived or patient-derived cancer cells cultivated and expanded on a perfusion microfluidic device (Figure 3). The chip allows the concurrent cultivation of living cells and ECM in a controllable environment and enables the establishment of a physiological concentration gradient of nutrient supply and waste removal using only picolitres to milliliters of solutions. Engineered tissue (multiple human cell types at physiologically relevant ratios) or patient-derived miniature tissues can grow inside the microfluidic chips and acquire the physiological properties of the source tumors [110,111].

Different available models recapitulate the tumor immunological landscape. For instance, BT474 or MCF7 breast cancer cells were placed with Hs578T cancer-associated fibroblasts and PBMCs in a chip together with a monolayer of endothelial cells (HUVECs) and used to study the role of CAF in invasion, immunomodulation, and immune antibody therapy [112].

Differently, Marzagalli et al. [21] exploited the high versatility of the tumor-on-a-chip technology and investigated the infiltration and cytotoxic activity of NK cells in a 3D model of neuroblastoma. In the same chip, tumor cells and immune cells cultivated in separate compartments were separated only by a porous membrane. After the application of fluid perfusion, they monitored the upstream extravasation of NK cells towards tumor cells. This model is of particular interest because it is suitable for testing immunotherapy in general and ICD-based therapy in particular. For instance, DC migration and activation experiments will produce more reliable results than the same experiments conducted in 2D cultures.

A more complex model was developed by Shireure et al. [113] and exploited by Bi et al. [114] to evaluate the role of macrophages during tumor progression. The chip consisted of three chambers separated from each other by porous membranes. Tumor cells with monocytes-derived macrophages (M1 or M2) were seeded on one side, while the central chamber was devoted to the formation of a perfused microvascular network by using a mixture of endothelial colony forming cell-derived endothelial cells and normal human lung fibroblasts in fibrin gel. The authors used this model to check the effect of M1 and M2 macrophages on tumor growth. Thus, it could be speculated that the same TME model can be exploited to evaluate and characterize the efficacy of ICD inducers in an immune-responsive (M1) or non-responsive (M2) TME.

A three-chamber chip was also used in a model that made it possible to evaluate immunotherapy by monitoring the behavior of dendritic cells towards colon cancer tumor cells. In this case, the central chamber held immature DC, while the side chambers held tumor cells embedded in type I collagen matrix to mimic the TME stromal part. On the one side, tumor cells treated with the anticancer agent romidepsin and INF-α2b were loaded, and untreated cells were loaded on the other side. An advanced microscopy platform was linked to the system and an algorithm allowed crucial immunogenic processes, such as cell–cell interactions, phagocytosis, and DC migration, to be monitored and analyzed [115].

Another three-chamber chip was used to engineer a microfluidic model of the tumor environment that can be used to study the effect of the TME on cellular drug responses in terms of cell death, oxidative stress, tumor proliferation and growth, and heterotypic cells interaction, such as immune cell migration. In this model, tumor cells were loaded in the central chamber within a collagen hydrogel mimicking the ECM, while the side chambers were used for perfusion or to add activated NK cells and examine their migration properties. Using fluorescence time-lapse microscopy, it was also possible to identify a necrotic core and to evaluate oxygen and glucose gradients within the tumor [116].

Organ-on-a-chip can be used to study biopsies from patients characterized by the presence of autologous immune cells (both lymphoid and myeloid subsets). To use these models, tumors must first be digested with collagenases and then loaded into the chip in a collagen matrix after allowing the formation of spheroids. In this model, several events linked to the immune response can be studied, such as cytokine release, profiling analysis, and efficacy of immunotherapy [117,118,119]. However, these responses are limited to pre-existing tumor-infiltrating immune cells and do not reflect the recruitment of additional immune cells into the model TME.

## 4. Future Perspective and Conclusions

It is well established that anticancer therapies, and particularly immunotherapies, strongly affect and are affected by the TME. Neoplastic lesions consist of tumor cells that are not isolated but exist within a stromal architecture made by cells, such as CAFs and endothelial cells, and the non-cellular matrix made of collagen or fibronectin. Immune cells of myeloid and lymphoid origin, such as macrophages and lymphocytes, cohabitate these structures and their status can dictate the final therapeutic outcome [18].

It is of note that only 3.4% of oncology clinical trials are successful and succeed in the third phase [120]. Most of the time, the lack of translational potential is due to the use of in vitro models that do not consider the large intra- and inter-tumor heterogenic composition to assess antitumor potential. In the context of immunotherapy and ICD, this issue is even more evident, and little information is currently known about the relationship between ICD and the TME. In this review, we describe different in vitro models that might be exploited to investigate the ICD potential of cytotoxic drugs and to study the overall effect of ICD within the TME (Figure 3). Every model has general advantages and disadvantages, which we can summarize by saying that the translational potential of each model is proportional to the complexity of the model, but managing the complexity of the model is not always possible and might require high expertise and considerable time and money to set up. For a more systematic and comprehensive analysis of each model mentioned above, we suggest reading some dedicated reviews, such as [21,22].

Two-dimensional models are the most common and easily operated models in the ICD field, pharmacology, and drug discovery (Figure 3). In terms of mirroring the TME, however, they have almost no resemblance. Still, they can be exploited to monitor the effect of ICD-dying cells on single tumor subpopulations. Indeed, studies on co-cultures of dying tumor cells and APCs provide an initial understanding of whether that cell death is immunogenic by monitoring the engulfment of dendritic cells (i.e., efferocytosis) or their activation and maturation. To better mirror the TME, hypoxic or acidic niches can be created by cultivating cancer cells in a hypoxic chamber or in an acidic medium. These conditions are often linked to therapeutic resistance or altered tumor cell behavior, so it is of interest to assess the ICD potential under these conditions. However, 2D models do not evaluate the effect of the stromal component or fully recapitulate the TME, and the degree of heterogenicity is very low. Theoretically, it would be possible to cultivate more than two cell types, but the unique distribution of the cells would make the model very artificial and not predictive.

To obviate this lack of predictivity, 3D models can be used because 3D cultures are more representative of the heterogenous features of solid tumors. Depending on the specific model, they produce their own ECM or can be embedded in a scaffold resembling the ECM. They can be formed from one or more cell types and can be derived from cell lines or patients’ biopsies. Indeed, the easiest 3D constructs are spheroids, which are more complex than flat 2D cultures because the spatial configuration allows cell–cell interaction and ECM production. As discussed above, the ECM is a key factor for cell differentiation, adhesion, migration, and survival. Spheroids can develop necrotic, hypoxic, or acidic cores, which can be exploited in ICD studies [45]. In addition, mono-cellular spheroids can be used to assess the potential of ICD inducers in a more relevant structure than in 2D cultures. Nevertheless, since no other cellular TME component is present, it is not possible to evaluate the ICD–TME interaction at a deeper level. So far, 3D spheroids have been used only to assess compounds’ ability to promote the activation of ICD markers, such as ER and oxidative stress, or DAMPs emission, or to study the effect of the co-cultivation of dying spheroids with DCs [121,122,123]. Furthermore, tumor spheroids can be used to check if the dying cells attract DCs after co-cultivation by monitoring the migration of the DCs towards the spheroid with confocal microscopy [124,125]. This would serve as an advanced classic Transwell migration assay to identify novel chemotactic molecules emitted by dying cancer cells.

In contrast to monotypic spheroids, heterotypic spheroids allow the inclusion of cellular components such as fibroblasts, CAFs or immune cells, and macrophages [20,60,63,126]. In this case, the spheroids are a better representation of the in vivo ECM, cell–cell contact, and cellular components other than tumor cells. Thus, they can be used to assess the efficacy of an ICD inducer by measuring cell death and immune cell activation as well as the effect of the ICD inducer on the different components of the TME.

Scaffold or bioprinted materials can be used to engineer organoids. Organoids consist of stem cells and differentiated cells resembling a tissue-like architecture, or they can be directly obtained from primary human tumor biopsies. These characteristics enable the realistic mirroring of tumor heterogenicity and can be used to investigate the interaction between the tumor and the TME, including immune cells. In particular, ICD studies can be performed after adding immune cells to organoids in a co-culture setting, as completed in 2D models and spheroids. In this case, an ICD inducer’s overall effect will be analyzed within a more elaborate TME and give more predictive outcomes. In addition, conceptually, organoids originating from primary tumors can be used. In this case, these models retain and facilitate the expansion of endogenous or non-endogenous immune cells, and can be exploited to assess the efficacy of ICD inducers. Nevertheless, one of the significant pitfalls of cultivating organoids is the short period during which the model keeps representing the original tumor. Indeed, organoids rarely maintain the original tumor genotype and phenotype and favor clonal tumor expansion, limiting tumor and TME heterogenicity [127]. Thus, this is an attractive mode to study ICD but it has also its own limitations.

Therefore, to overcome this issue, more representative TME can be obtained by installing perfusion systems to add flow to the system. Perfusion bioreactors and organs-on-a-chip are two examples. In organoids, tumors are located on scaffolds that mimic the ECM, and a tube/microtube system maintains nutrient and oxygenation gradients and waste removal. For perfusion models too, tumors can be installed using human/mouse ex vivo biopsies or a mixture of different cell types, including tumor and immune cells. The main advantage of perfusion is that it guarantees the long-term cultivation of neoplastic lesions and preserves genetic and phenotypic signatures. Although very few studies have used these models to analyze ICD, in that context, the perfusion system is an added value because after the treatment of cancer cells and their migration, DAMPs or cytokine release can be easily monitored.

What all perfusion models have in common is that they can be engineered by choosing tumor and immune cells (including cell lines or single cell types, such as tumor cells or human PBMCs) or exploiting heterogenous tumor samples from patients. The choice of the model depends on the researcher’s aim. For basic ICD studies, the high complexity of the ex vivo samples might make mechanistic studies difficult. Nevertheless, that same complexity is optimal for testing the efficacy of ICD inducers in terms of cytotoxicity and immune cell activation and monitoring patient-to-patient variations. For instance, compartmentalization in different chambers of an organ-on-a-chip has been used to demonstrate that MDA-MB-231 breast cancer cells treated with the ICD inducers doxorubicin and mitoxantrone promote PBMC migration towards the tumor cells [128]. However, the same assay would be harder to perform on ex vivo samples in terms of the general signal-to-noise and procedure type and length. Nonetheless, in the near future, the organ-on-a-chip technology will allow more complex experiments, including the use of both tumor explants and full patient-derived organs in the same device. For instance, in 2021, the EU commission funded a project that aims to engineer an automated tumor–lymph node-on-chip platform that can connect primary tumors to lymph node tissue obtained from the same cancer patient [129]. This model would be of great interest for ICD research. In fact, this technology might also overcome one of the most serious limitations of using samples obtained from patients, which is the inclusion of only immune cells already within the tumor (Figure 3). The presence of an autologous lymph node would allow the evaluation of both humoral and cell-mediated immune responses upon treatment with the ICD inducer. Theoretically, this model would make it possible to conduct in vitro experiments as a sort of surrogate of vaccination. This would be of great importance for several reasons. First, the latter-mentioned tumor–organ–chip is ethically desirable because it would reduce the number of animals used for research, as the only way so far to assess whether an ICD candidate triggers an adaptive immune response is using mouse models (prophylactic vaccination or abscopal therapeutic models) [130]). Furthermore, although mice are routinely used to model human diseases, their immune system differs from that of humans, and these differences could be a reason for the high rate of failure of clinical trials. Thus, this chip will outstandingly increase the translatability of ICD testing.

While waiting for this technology to be finalized, primary tumors can still be cultivated in perfusion bioreactors/organs-on-a-chip and used to develop targeted therapy. As mentioned above, tumor samples from patients comprise all the complexity of the in vivo TME, which is crucial to consider when evaluating the efficacy of ICD inducers; bioreactors preserve this complexity. Bioreactors may be used to test and identify hot and cold tumors, and thus responding and non-responding patients. Furthermore, it is known that ICD inducers modify the antigen landscape of tumors [131], and perfusion bioreactors might be used as a platform to therapeutically induce the formation of new antigens. The identification of those specific antigens and their loading onto autologous DC can become the rationale for the preparation of personalized anticancer vaccines. Although this strategy is far from achievable, it shows the potential of perfusion bioreactors in the ICD context.

In conclusion, one of the major limitations of immune anticancer research is the lack of translational potential of the conventional in vitro models, which insufficiently mimic the complex immunobiology of native human tumors. Including the TME in the analysis and increasing cell heterogenicity of the model are two crucial factors that should be taken into consideration. Nevertheless, ICD assays should be implemented in the available models, such as 2D and mono- and heterocellular spheroids. Research efforts should be directed to develop new models and improve the utility of current complex models since they are characterized by a higher translational potential (Figure 3). This is a challenging area for future research and will bring new insights into the molecular mechanisms of ICD in the context of TME and lead to novel experimental immunotherapies for cancer.

## Figures and Tables

**Table 1:** Advantages and disadvantages of 2D TME models and assays.

Model	Advantages	Disadvantages
Phagocytosis (efferocytosis)	Direct evidence of interaction and/or dying/dead cancer cell engulfment	Limited (to the surface area and cell variety) and artificial environment of the phagocytosis assay (culture media concentrated with SNs of the dying/dead cancer cells). Highly dependent on the ratios of dead/dying cells towards the APCs and time of their co-culture.
Maturation/activation of APCs Polarization of macrophages	Direct evidence (on morphological, genetic, protein, and physiological levels) of immune response towards the (treated) dying/dead cancer cells	Similar to the phagocytosis.
Alteration of cancer cell metabolism	Closer recreation of TME conditions (low glucose, hypoxia, etc.)	Time- (chronic exposure of cells is needed to switch to hypoxic/acidic metabolism) and labor-intensive (special amino and fatty acids with deprived or conditioned media).

## Data Availability

Not applicable.

## References

[1] Galluzzi L., Buqué A., Kepp O., Zitvogel L., Kroemer G. (2017). Immunogenic Cell Death in Cancer and Infectious Disease. Nat. Rev. Immunol..

[2] Krysko D.V., Garg A.D., Kaczmarek A., Krysko O., Agostinis P., Vandenabeele P. (2012). Immunogenic Cell Death and DAMPs in Cancer Therapy. Nat. Rev. Cancer.

[3] Fucikova J., Kepp O., Kasikova L., Petroni G., Yamazaki T., Liu P., Zhao L., Spisek R., Kroemer G., Galluzzi L. (2020). Detection of Immunogenic Cell Death and Its Relevance for Cancer Therapy. Cell Death Dis..

[4] Casares N., Pequignot M.O., Tesniere A., Ghiringhelli F., Roux S., Chaput N., Schmitt E., Hamai A., Hervas-Stubbs S., Obeid M. (2005). Caspase-Dependent Immunogenicity of Doxorubicin-Induced Tumor Cell Death. J. Exp. Med..

[5] Kroemer G., Galassi C., Zitvogel L., Galluzzi L. (2022). Immunogenic Cell Stress and Death. Nat. Immunol..

[6] Garg A.D., Krysko D.V., Verfaillie T., Kaczmarek A., Ferreira G.B., Marysael T., Rubio N., Firczuk M., Mathieu C., Roebroek A.J.M. (2012). A Novel Pathway Combining Calreticulin Exposure and ATP Secretion in Immunogenic Cancer Cell Death. EMBO J..

[7] Efimova I., Catanzaro E., Van der Meeren L., Turubanova V.D., Hammad H., Mishchenko T.A., Vedunova M.V., Fimognari C., Bachert C., Coppieters F. (2020). Vaccination with Early Ferroptotic Cancer Cells Induces Efficient Antitumor Immunity. J. Immunother. Cancer.

[8] Liu J., Zhu S., Zeng L., Li J., Klionsky D.J., Kroemer G., Jiang J., Tang D., Kang R. (2022). DCN Released from Ferroptotic Cells Ignites AGER-Dependent Immune Responses. Autophagy.

[9] Aaes T.L., Kaczmarek A., Delvaeye T., De Craene B., De Koker S., Heyndrickx L., Delrue I., Taminau J., Wiernicki B., De Groote P. (2016). Vaccination with Necroptotic Cancer Cells Induces Efficient Anti-Tumor Immunity. Cell Rep..

[10] Yatim N., Jusforgues-Saklani H., Orozco S., Schulz O., Barreira da Silva R., Reis e Sousa C., Green D.R., Oberst A., Albert M.L. (2015). RIPK1 and NF-ΚB Signaling in Dying Cells Determines Cross-Priming of CD8^+^ T Cells. Science.

[11] Turubanova V.D., Mishchenko T.A., Balalaeva I.V., Efimova I., Peskova N.N., Klapshina L.G., Lermontova S.A., Bachert C., Krysko O., Vedunova M.V. (2021). Novel Porphyrazine-Based Photodynamic Anti-Cancer Therapy Induces Immunogenic Cell Death. Sci. Rep..

[12] Bloy N., Garcia P., Laumont C.M., Pitt J.M., Sistigu A., Stoll G., Yamazaki T., Bonneil E., Buqué A., Humeau J. (2017). Immunogenic Stress and Death of Cancer Cells: Contribution of Antigenicity vs Adjuvanticity to Immunosurveillance. Immunol. Rev..

[13] Galluzzi L., Petroni G., Kroemer G. (2020). Immunogenicity of Cell Death Driven by Immune Effectors. J. Immunother. Cancer.

[14] Rufo N., Garg A.D., Agostinis P. (2017). The Unfolded Protein Response in Immunogenic Cell Death and Cancer Immunotherapy. Trends Cancer.

[15] Bezu L., Sauvat A., Humeau J., Gomes-da-Silva L.C., Iribarren K., Forveille S., Garcia P., Zhao L., Liu P., Zitvogel L. (2018). EIF2α Phosphorylation Is Pathognomonic for Immunogenic Cell Death. Cell Death Differ..

[16] Garg A.D., More S., Rufo N., Mece O., Sassano M.L., Agostinis P., Zitvogel L., Kroemer G., Galluzzi L. (2017). Trial Watch: Immunogenic Cell Death Induction by Anticancer Chemotherapeutics. Oncoimmunology.

[17] Demuynck R., Efimova I., Naessens F., Krysko D.V. (2021). Immunogenic Ferroptosis and Where to Find It?. J. Immunother. Cancer.

[18] Anderson N.M., Simon M.C. (2020). The Tumor Microenvironment. Curr. Biol. CB.

[19] Hinshaw D.C., Shevde L.A. (2019). The Tumor Microenvironment Innately Modulates Cancer Progression. Cancer Res..

[20] Hanahan D. (2022). Hallmarks of Cancer: New Dimensions. Cancer Discov..

[21] Marzagalli M., Pelizzoni G., Fedi A., Vitale C., Fontana F., Bruno S., Poggi A., Dondero A., Aiello M., Castriconi R. (2022). A Multi-Organ-on-Chip to Recapitulate the Infiltration and the Cytotoxic Activity of Circulating NK Cells in 3D Matrix-Based Tumor Model. Front. Bioeng. Biotechnol..

[22] Fitzgerald A.A., Li E., Weiner L.M. (2021). 3D Culture Systems for Exploring Cancer Immunology. Cancers.

[23] Krysko D.V., Vandenabeele P. (2010). Clearance of Dead Cells: Mechanisms, Immune Responses and Implication in the Development of Diseases. Apoptosis Int. J. Program. Cell Death.

[24] Krysko D.V., Brouckaert G., Kalai M., Vandenabeele P., D’Herde K. (2003). Mechanisms of Internalization of Apoptotic and Necrotic L929 Cells by a Macrophage Cell Line Studied by Electron Microscopy. J. Morphol..

[25] Krysko D.V., Denecker G., Festjens N., Gabriels S., Parthoens E., D’Herde K., Vandenabeele P. (2006). Macrophages Use Different Internalization Mechanisms to Clear Apoptotic and Necrotic Cells. Cell Death Differ..

[26] Lin J., Xu A., Jin J., Zhang M., Lou J., Qian C., Zhu J., Wang Y., Yang Z., Li X. (2022). MerTK-Mediated Efferocytosis Promotes Immune Tolerance and Tumor Progression in Osteosarcoma through Enhancing M2 Polarization and PD-L1 Expression. OncoImmunology.

[27] Werfel T.A., Elion D.L., Rahman B., Hicks D.J., Sanchez V., Gonzales-Ericsson P.I., Nixon M.J., James J.L., Balko J.M., Scherle P.A. (2019). Treatment-Induced Tumor Cell Apoptosis and Secondary Necrosis Drive Tumor Progression in the Residual Tumor Microenvironment through MERTK and IDO1. Cancer Res..

[28] Wu C., Tan X., Hu X., Zhou M., Yan J., Ding C. (2020). Tumor Microenvironment Following Gemcitabine Treatment Favors Differentiation of Immunosuppressive Ly6Chigh Myeloid Cells. J. Immunol..

[29] Petty A.J., Li A., Wang X., Dai R., Heyman B., Hsu D., Huang X., Yang Y. (2019). Hedgehog Signaling Promotes Tumor-Associated Macrophage Polarization to Suppress Intratumoral CD8+ T Cell Recruitment. J. Clin. Investig..

[30] Li Q., Ma L., Shen S., Guo Y., Cao Q., Cai X., Feng J., Yan Y., Hu T., Luo S. (2019). Intestinal Dysbacteriosis-Induced IL-25 Promotes Development of HCC via Alternative Activation of Macrophages in Tumor Microenvironment. J. Exp. Clin. Cancer Res..

[31] Chen X.J., Wu S., Yan R.M., Fan L.S., Yu L., Zhang Y.M., Wei W.F., Zhou C.F., Wu X.G., Zhong M. (2019). The Role of the Hypoxia-Nrp-1 Axis in the Activation of M2-like Tumor-Associated Macrophages in the Tumor Microenvironment of Cervical Cancer. Mol. Carcinog..

[32] Lee H.E., Lee J.Y., Yang G., Kang H.C., Cho Y.Y., Lee H.S., Lee J.Y. (2019). Inhibition of NLRP3 Inflammasome in Tumor Microenvironment Leads to Suppression of Metastatic Potential of Cancer Cells. Sci. Rep..

[33] Lauber K., Bohn E., Kröber S.M., Xiao Y., Blumenthal S.G., Lindemann R.K., Marini P., Wiedig C., Zobywalski A., Baksh S. (2003). Apoptotic Cells Induce Migration of Phagocytes via Caspase-3-Mediated Release of a Lipid Attraction Signal. Cell.

[34] Andreucci E., Peppicelli S., Ruzzolini J., Bianchini F., Biagioni A., Papucci L., Magnelli L., Mazzanti B., Stecca B., Calorini L. (2020). The Acidic Tumor Microenvironment Drives a Stem-like Phenotype in Melanoma Cells. J. Mol. Med..

[35] Mathews E.H., Visagie M.H., Meyer A.A., Joubert A.M., Mathews G.E. (2020). In Vitro Quantification: Long-Term Effect of Glucose Deprivation on Various Cancer Cell Lines. Nutrition.

[36] Horsman M.R., Vaupel P. (2016). Pathophysiological Basis for the Formation of the Tumor Microenvironment. Front. Oncol..

[37] Jing X., Yang F., Shao C., Wei K., Xie M., Shen H., Shu Y. (2019). Role of Hypoxia in Cancer Therapy by Regulating the Tumor Microenvironment. Mol. Cancer.

[38] Sudhan D.R., Siemann D.W. (2013). Cathepsin L Inhibition by the Small Molecule KGP94 Suppresses Tumor Microenvironment Enhanced Metastasis Associated Cell Functions of Prostate and Breast Cancer Cells. Clin. Exp. Metastasis.

[39] Place T.L., Domann F.E., Case A.J. (2017). Limitations of Oxygen Delivery to Cells in Culture: An Underappreciated Problem in Basic and Translational Research. Free Radic. Biol. Med..

[40] Campillo N., Falcones B., Otero J., Colina R., Gozal D., Navajas D., Farré R., Almendros I. (2019). Differential Oxygenation in Tumor Microenvironment Modulates Macrophage and Cancer Cell Crosstalk: Novel Experimental Settingand Proof of Concept. Front. Oncol..

[41] Pavlacky J., Polak J. (2020). Technical Feasibility and Physiological Relevance of Hypoxic Cell Culture Models. Front. Endocrinol..

[42] Riemann A., Schneider B., Gündel D., Stock C., Gekle M., Thews O. (2016). Acidosis Promotes Metastasis Formation by Enhancing Tumor Cell Motility. Adv. Exp. Med. Biol..

[43] Rohani N., Hao L., Alexis M.S., Joughin B.A., Krismer K., Moufarrej M.N., Soltis A.R., Lauffenburger D.A., Yaffe M.B., Burge C.B. (2019). Acidification of Tumor at Stromal Boundaries Drives Transcriptome Alterations Associated with Aggressive Phenotypes. Cancer Res..

[44] Rauschner M., Hüsing T., Lange L., Jarosik K., Reime S., Riemann A., Thews O. (2021). Role of Acidosis-Sensitive MicroRNAs in Gene Expression and Functional Parameters of Tumors in Vitro and in Vivo. Neoplasia.

[45] Dierge E., Debock E., Guilbaud C., Corbet C., Mignolet E., Mignard L., Bastien E., Dessy C., Larondelle Y., Feron O. (2021). Peroxidation of N-3 and n-6 Polyunsaturated Fatty Acids in the Acidic Tumor Environment Leads to Ferroptosis-Mediated Anticancer Effects. Cell Metab..

[46] Khajah M.A., Khushaish S., Luqmani Y.A. (2022). Glucose Deprivation Reduces Proliferation and Motility, and Enhances the Anti-Proliferative Effects of Paclitaxel and Doxorubicin in Breast Cell Lines in Vitro. PLoS ONE.

[47] Zhang M., Liu T., Sun H., Weng W., Zhang Q., Liu C., Han Y., Sheng W. (2018). Pim1 Supports Human Colorectal Cancer Growth during Glucose Deprivation by Enhancing the Warburg Effect. Cancer Sci..

[48] Demuynck R., Efimova I., Lin A., Declercq H., Krysko D.V. (2020). A 3D Cell Death Assay to Quantitatively Determine Ferroptosis in Spheroids. Cells.

[49] Meyenberg Cunha-de Padua M., Noleto G.R., de Oliveira Petkowicz C.L., Cadena S.M.S.C., Bost F., Pouysségur J., Mazure N.M. (2019). Hypoxia Protects against the Cell Death Triggered by Oxovanadium-Galactomannan Complexes in HepG2 Cells. Cell. Mol. Biol. Lett..

[50] Jensen C., Teng Y. (2020). Is It Time to Start Transitioning From 2D to 3D Cell Culture?. Front. Mol. Biosci..

[51] Xu S., Yang Y., Dong L., Qiu W., Yang L., Wang X., Liu L. (2014). Construction and Characteristics of an E-Cadherin-Related Three-Dimensional Suspension Growth Model of Ovarian Cancer. Sci. Rep..

[52] Gong X., Lin C., Cheng J., Su J., Zhao H., Liu T., Wen X., Zhao P. (2015). Generation of Multicellular Tumor Spheroids with Microwell-Based Agarose Scaffolds for Drug Testing. PLoS ONE.

[53] Yang X., Sarvestani S.K., Moeinzadeh S., He X., Jabbari E. (2013). Effect of CD44 Binding Peptide Conjugated to an Engineered Inert Matrix on Maintenance of Breast Cancer Stem Cells and Tumorsphere Formation. PLoS ONE.

[54] Peirsman A., Blondeel E., Ahmed T., Anckaert J., Audenaert D., Boterberg T., Buzas K., Carragher N., Castellani G., Castro F. (2021). MISpheroID: A Knowledgebase and Transparency Tool for Minimum Information in Spheroid Identity. Nat. Methods.

[55] Ryu N.-E., Lee S.-H., Park H. (2019). Spheroid Culture System Methods and Applications for Mesenchymal Stem Cells. Cells.

[56] Longati P., Jia X., Eimer J., Wagman A., Witt M.-R., Rehnmark S., Verbeke C., Toftgård R., Löhr M., Heuchel R.L. (2013). 3D Pancreatic Carcinoma Spheroids Induce a Matrix-Rich, Chemoresistant Phenotype Offering a Better Model for Drug Testing. BMC Cancer.

[57] Zhao L., Xiu J., Liu Y., Zhang T., Pan W., Zheng X., Zhang X. (2019). A 3D Printed Hanging Drop Dripper for Tumor Spheroids Analysis Without Recovery. Sci. Rep..

[58] Jeong S.-Y., Lee J.-H., Shin Y., Chung S., Kuh H.-J. (2016). Co-Culture of Tumor Spheroids and Fibroblasts in a Collagen Matrix-Incorporated Microfluidic Chip Mimics Reciprocal Activation in Solid Tumor Microenvironment. PLoS ONE.

[59] Santos J.M., Camões S.P., Filipe E., Cipriano M., Barcia R.N., Filipe M., Teixeira M., Simões S., Gaspar M., Mosqueira D. (2015). Three-Dimensional Spheroid Cell Culture of Umbilical Cord Tissue-Derived Mesenchymal Stromal Cells Leads to Enhanced Paracrine Induction of Wound Healing. Stem Cell Res. Ther..

[60] De Moor L., Merovci I., Baetens S., Verstraeten J., Kowalska P., Krysko D.V., De Vos W.H., Declercq H. (2018). High-Throughput Fabrication of Vascularized Spheroids for Bioprinting. Biofabrication.

[61] Stadler M., Scherzer M., Walter S., Holzner S., Pudelko K., Riedl A., Unger C., Kramer N., Weil B., Neesen J. (2018). Exclusion from Spheroid Formation Identifies Loss of Essential Cell-Cell Adhesion Molecules in Colon Cancer Cells. Sci. Rep..

[62] Bai C., Yang M., Fan Z., Li S., Gao T., Fang Z. (2015). Associations of Chemo- and Radio-Resistant Phenotypes with the Gap Junction, Adhesion and Extracellular Matrix in a Three-Dimensional Culture Model of Soft Sarcoma. J. Exp. Clin. Cancer Res..

[63] Bulin A.-L., Broekgaarden M., Simeone D., Hasan T. (2019). Low Dose Photodynamic Therapy Harmonizes with Radiation Therapy to Induce Beneficial Effects on Pancreatic Heterocellular Spheroids. Oncotarget.

[64] Rebelo S.P., Pinto C., Martins T.R., Harrer N., Estrada M.F., Loza-Alvarez P., Cabeçadas J., Alves P.M., Gualda E.J., Sommergruber W. (2018). 3D-3-Culture: A Tool to Unveil Macrophage Plasticity in the Tumour Microenvironment. Biomaterials.

[65] Lee Y.B., Kim E.M., Byun H., Chang H., Jeong K., Aman Z.M., Choi Y.S., Park J., Shin H. (2018). Engineering Spheroids Potentiating Cell-Cell and Cell-ECM Interactions by Self-Assembly of Stem Cell Microlayer. Biomaterials.

[66] Aoudjit F., Vuori K. (2012). Integrin Signaling in Cancer Cell Survival and Chemoresistance. Chemother. Res. Pract..

[67] Chang Q., Ornatsky O.I., Siddiqui I., Straus R., Baranov V.I., Hedley D.W. (2016). Biodistribution of Cisplatin Revealed by Imaging Mass Cytometry Identifies Extensive Collagen Binding in Tumor and Normal Tissues. Sci. Rep..

[68] Tan Y., Tajik A., Chen J., Jia Q., Chowdhury F., Wang L., Chen J., Zhang S., Hong Y., Yi H. (2014). Matrix Softness Regulates Plasticity of Tumour-Repopulating Cells via H3K9 Demethylation and Sox2 Expression. Nat. Commun..

[69] Jang M., Koh I., Lee J.E., Lim J.Y., Cheong J.-H., Kim P. (2018). Increased Extracellular Matrix Density Disrupts E-Cadherin/β-Catenin Complex in Gastric Cancer Cells. Biomater. Sci..

[70] Pang M.-F., Siedlik M.J., Han S., Stallings-Mann M., Radisky D.C., Nelson C.M. (2016). Tissue Stiffness and Hypoxia Modulate the Integrin-Linked Kinase ILK to Control Breast Cancer Stem-like Cells. Cancer Res..

[71] Garcia-Mayea Y., Mir C., Masson F., Paciucci R., LLeonart M.E. (2020). Insights into New Mechanisms and Models of Cancer Stem Cell Multidrug Resistance. Semin. Cancer Biol..

[72] Huang Q., Zou Y., Arno M.C., Chen S., Wang T., Gao J., Dove A.P., Du J. (2017). Hydrogel Scaffolds for Differentiation of Adipose-Derived Stem Cells. Chem. Soc. Rev..

[73] Ricard-Blum S. (2011). The Collagen Family. Cold Spring Harb. Perspect. Biol..

[74] Kuczek D.E., Larsen A.M.H., Thorseth M.-L., Carretta M., Kalvisa A., Siersbæk M.S., Simões A.M.C., Roslind A., Engelholm L.H., Noessner E. (2019). Collagen Density Regulates the Activity of Tumor-Infiltrating T Cells. J. Immunother. Cancer.

[75] Van Hoorick J., Gruber P., Markovic M., Tromayer M., Van Erps J., Thienpont H., Liska R., Ovsianikov A., Dubruel P., Van Vlierberghe S. (2017). Cross-Linkable Gelatins with Superior Mechanical Properties Through Carboxylic Acid Modification: Increasing the Two-Photon Polymerization Potential. Biomacromolecules.

[76] Augustine R., Zahid A.A., Mraiche F., Alam K., Al Moustafa A.-E., Hasan A. (2021). Gelatin-Methacryloyl Hydrogel Based in Vitro Blood-Brain Barrier Model for Studying Breast Cancer-Associated Brain Metastasis. Pharm. Dev. Technol..

[77] Hughes C.S., Postovit L.M., Lajoie G.A. (2010). Matrigel: A Complex Protein Mixture Required for Optimal Growth of Cell Culture. Proteomics.

[78] Keeratichamroen S., Lirdprapamongkol K., Svasti J. (2018). Mechanism of ECM-Induced Dormancy and Chemoresistance in A549 Human Lung Carcinoma Cells. Oncol. Rep..

[79] Edmondson R., Adcock A.F., Yang L. (2016). Influence of Matrices on 3D-Cultured Prostate Cancer Cells’ Drug Response and Expression of Drug-Action Associated Proteins. PLoS ONE.

[80] Sodek K.L., Brown T.J., Ringuette M.J. (2008). Collagen I but Not Matrigel Matrices Provide an MMP-Dependent Barrier to Ovarian Cancer Cell Penetration. BMC Cancer.

[81] Anguiano M., Morales X., Castilla C., Pena A.R., Ederra C., Martínez M., Ariz M., Esparza M., Amaveda H., Mora M. (2020). The Use of Mixed Collagen-Matrigel Matrices of Increasing Complexity Recapitulates the Biphasic Role of Cell Adhesion in Cancer Cell Migration: ECM Sensing, Remodeling and Forces at the Leading Edge of Cancer Invasion. PLoS ONE.

[82] Cavo M., Caria M., Pulsoni I., Beltrame F., Fato M., Scaglione S. (2018). A New Cell-Laden 3D Alginate-Matrigel Hydrogel Resembles Human Breast Cancer Cell Malignant Morphology, Spread and Invasion Capability Observed “In Vivo”. Sci. Rep..

[83] De Jaeghere E., De Vlieghere E., Van Hoorick J., Van Vlierberghe S., Wagemans G., Pieters L., Melsens E., Praet M., Van Dorpe J., Boone M.N. (2018). Heterocellular 3D Scaffolds as Biomimetic to Recapitulate the Tumor Microenvironment of Peritoneal Metastases in Vitro and in Vivo. Biomaterials.

[84] Nanki Y., Chiyoda T., Hirasawa A., Ookubo A., Itoh M., Ueno M., Akahane T., Kameyama K., Yamagami W., Kataoka F. (2020). Patient-Derived Ovarian Cancer Organoids Capture the Genomic Profiles of Primary Tumours Applicable for Drug Sensitivity and Resistance Testing. Sci. Rep..

[85] Múnera J.O., Wells J.M. (2017). Generation of Gastrointestinal Organoids from Human Pluripotent Stem Cells. Methods Mol. Biol. Clifton.

[86] Voabil P., de Bruijn M., Roelofsen L.M., Hendriks S.H., Brokamp S., van den Braber M., Broeks A., Sanders J., Herzig P., Zippelius A. (2021). An Ex Vivo Tumor Fragment Platform to Dissect Response to PD-1 Blockade in Cancer. Nat. Med..

[87] Yan H.H.N., Siu H.C., Law S., Ho S.L., Yue S.S.K., Tsui W.Y., Chan D., Chan A.S., Ma S., Lam K.O. (2018). A Comprehensive Human Gastric Cancer Organoid Biobank Captures Tumor Subtype Heterogeneity and Enables Therapeutic Screening. Cell Stem Cell.

[88] Kucab J.E., Hollstein M., Arlt V.M., Phillips D.H. (2017). Nutlin-3a Selects for Cells Harbouring TP53 Mutations. Int. J. Cancer.

[89] Lee S.H., Hu W., Matulay J.T., Silva M.V., Owczarek T.B., Kim K., Chua C.W., Barlow L.J., Kandoth C., Williams A.B. (2018). Tumor Evolution and Drug Response in Patient-Derived Organoid Models of Bladder Cancer. Cell.

[90] Nuciforo S., Fofana I., Matter M.S., Blumer T., Calabrese D., Boldanova T., Piscuoglio S., Wieland S., Ringnalda F., Schwank G. (2018). Organoid Models of Human Liver Cancers Derived from Tumor Needle Biopsies. Cell Rep..

[91] Shiihara M., Furukawa T. (2022). Application of Patient-Derived Cancer Organoids to Personalized Medicine. J. Pers. Med..

[92] Cattaneo C.M., Dijkstra K.K., Fanchi L.F., Kelderman S., Kaing S., van Rooij N., van den Brink S., Schumacher T.N., Voest E.E. (2020). Tumor Organoid-T-Cell Coculture Systems. Nat. Protoc..

[93] Valančiūtė A., Mathieson L., O’Connor R.A., Scott J.I., Vendrell M., Dorward D.A., Akram A.R., Dhaliwal K. (2022). Phototherapeutic Induction of Immunogenic Cell Death and CD8+ T Cell-Granzyme B Mediated Cytolysis in Human Lung Cancer Cells and Organoids. Cancers.

[94] Duval K., Grover H., Han L.-H., Mou Y., Pegoraro A.F., Fredberg J., Chen Z. (2017). Modeling Physiological Events in 2D vs. 3D Cell Culture. Physiol. Bethesda Md.

[95] Muraro M.G., Muenst S., Mele V., Quagliata L., Iezzi G., Tzankov A., Weber W.P., Spagnoli G.C., Soysal S.D. (2017). Ex-Vivo Assessment of Drug Response on Breast Cancer Primary Tissue with Preserved Microenvironments. Oncoimmunology.

[96] Hirt C., Papadimitropoulos A., Muraro M.G., Mele V., Panopoulos E., Cremonesi E., Ivanek R., Schultz-Thater E., Droeser R.A., Mengus C. (2015). Bioreactor-Engineered Cancer Tissue-like Structures Mimic Phenotypes, Gene Expression Profiles and Drug Resistance Patterns Observed “In Vivo”. Biomaterials.

[97] Manfredonia C., Muraro M.G., Hirt C., Mele V., Governa V., Papadimitropoulos A., Däster S., Soysal S.D., Droeser R.A., Mechera R. (2019). Maintenance of Primary Human Colorectal Cancer Microenvironment Using a Perfusion Bioreactor-Based 3D Culture System. Adv. Biosyst..

[98] Goliwas K.F., Marshall L.E., Ransaw E.L., Berry J.L., Frost A.R. (2016). A Recapitulative Three-Dimensional Model of Breast Carcinoma Requires Perfusion for Multi-Week Growth. J. Tissue Eng..

[99] Pasini A., Lovecchio J., Cortesi M., Liverani C., Spadazzi C., Mercatali L., Ibrahim T., Giordano E. (2021). Perfusion Flow Enhances Viability and Migratory Phenotype in 3D-Cultured Breast Cancer Cells. Ann. Biomed. Eng..

[100] Rafaeva M., Horton E.R., Jensen A.R.D., Madsen C.D., Reuten R., Willacy O., Brøchner C.B., Jensen T.H., Zornhagen K.W., Crespo M. (2022). Modeling Metastatic Colonization in a Decellularized Organ Scaffold-Based Perfusion Bioreactor. Adv. Healthc. Mater..

[101] Martinez A., Buckley M.S., Scalise C.B., Wang D., Katre A.A., Birrer M.J., Berry J.L., Arend R.C. (2021). Utilization of a 3-D Tissue Engineered Model to Investigate the Effects of Perfusion on Gynecologic Cancer Biology. J. Tissue Eng..

[102] Jasuja H., Kar S., Katti D.R., Katti K.S. (2021). Perfusion Bioreactor Enabled Fluid-Derived Shear Stress Conditions for Novel Bone Metastatic Prostate Cancer Testbed. Biofabrication.

[103] Trachtenberg J.E., Santoro M., Williams C., Piard C.M., Smith B.T., Placone J.K., Menegaz B.A., Molina E.R., Lamhamedi-Cherradi S.-E., Ludwig J.A. (2018). Effects of Shear Stress Gradients on Ewing Sarcoma Cells Using 3D Printed Scaffolds and Flow Perfusion. ACS Biomater. Sci. Eng..

[104] Santoro M., Lamhamedi-Cherradi S.-E., Menegaz B.A., Ludwig J.A., Mikos A.G. (2015). Flow Perfusion Effects on Three-Dimensional Culture and Drug Sensitivity of Ewing Sarcoma. Proc. Natl. Acad. Sci. USA.

[105] Usuludin S.B.M., Cao X., Lim M. (2012). Co-Culture of Stromal and Erythroleukemia Cells in a Perfused Hollow Fiber Bioreactor System as an in Vitro Bone Marrow Model for Myeloid Leukemia. Biotechnol. Bioeng..

[106] García-García A., Klein T., Born G., Hilpert M., Scherberich A., Lengerke C., Skoda R.C., Bourgine P.E., Martin I. (2021). Culturing Patient-Derived Malignant Hematopoietic Stem Cells in Engineered and Fully Humanized 3D Niches. Proc. Natl. Acad. Sci. USA.

[107] Shekarian T., Zinner C.P., Bartoszek E.M., Duchemin W., Wachnowicz A.T., Hogan S., Etter M.M., Flammer J., Paganetti C., Martins T.A. (2022). Immunotherapy of Glioblastoma Explants Induces Interferon-γ Responses and Spatial Immune Cell Rearrangements in Tumor Center, but Not Periphery. Sci. Adv..

[108] Di Maggio N., Piccinini E., Jaworski M., Trumpp A., Wendt D.J., Martin I. (2011). Toward Modeling the Bone Marrow Niche Using Scaffold-Based 3D Culture Systems. Biomaterials.

[109] Guller A.E., Grebenyuk P.N., Shekhter A.B., Zvyagin A.V., Deyev S.M. (2016). Bioreactor-Based Tumor Tissue Engineering. Acta Naturae.

[110] Leung C.M., de Haan P., Ronaldson-Bouchard K., Kim G.-A., Ko J., Rho H.S., Chen Z., Habibovic P., Jeon N.L., Takayama S. (2022). A Guide to the Organ-on-a-Chip. Nat. Rev. Methods Primer.

[111] Yoon P.S., Del Piccolo N., Shirure V.S., Peng Y., Kirane A., Canter R.J., Fields R.C., George S.C., Gholami S. (2020). Advances in Modeling the Immune Microenvironment of Colorectal Cancer. Front. Immunol..

[112] Nguyen M., De Ninno A., Mencattini A., Mermet-Meillon F., Fornabaio G., Evans S.S., Cossutta M., Khira Y., Han W., Sirven P. (2018). Dissecting Effects of Anti-Cancer Drugs and Cancer-Associated Fibroblasts by On-Chip Reconstitution of Immunocompetent Tumor Microenvironments. Cell Rep..

[113] Shirure V.S., Bi Y., Curtis M.B., Lezia A., Goedegebuure M.M., Goedegebuure S.P., Aft R., Fields R.C., George S.C. (2018). Tumor-on-a-Chip Platform to Investigate Progression and Drug Sensitivity in Cell Lines and Patient-Derived Organoids. Lab. Chip.

[114] Bi Y., Shirure V.S., Liu R., Cunningham C., Ding L., Meacham J.M., Goedegebuure S.P., George S.C., Fields R.C. (2020). Tumor-on-a-Chip Platform to Interrogate the Role of Macrophages in Tumor Progression. Integr. Biol..

[115] Parlato S., De Ninno A., Molfetta R., Toschi E., Salerno D., Mencattini A., Romagnoli G., Fragale A., Roccazzello L., Buoncervello M. (2017). 3D Microfluidic Model for Evaluating Immunotherapy Efficacy by Tracking Dendritic Cell Behaviour toward Tumor Cells. Sci. Rep..

[116] Ayuso J.M., Virumbrales-Muñoz M., Lacueva A., Lanuza P.M., Checa-Chavarria E., Botella P., Fernández E., Doblare M., Allison S.J., Phillips R.M. (2016). Development and Characterization of a Microfluidic Model of the Tumour Microenvironment. Sci. Rep..

[117] Jenkins R.W., Aref A.R., Lizotte P.H., Ivanova E., Stinson S., Zhou C.W., Bowden M., Deng J., Liu H., Miao D. (2018). Ex Vivo Profiling of PD-1 Blockade Using Organotypic Tumor Spheroids. Cancer Discov..

[118] Deng J., Wang E.S., Jenkins R.W., Li S., Dries R., Yates K., Chhabra S., Huang W., Liu H., Aref A.R. (2018). CDK4/6 Inhibition Augments Antitumor Immunity by Enhancing T-Cell Activation. Cancer Discov..

[119] Aref A.R., Campisi M., Ivanova E., Portell A., Larios D., Piel B.P., Mathur N., Zhou C., Coakley R.V., Bartels A. (2018). 3D Microfluidic Ex Vivo Culture of Organotypic Tumor Spheroids to Model Immune Checkpoint Blockade. Lab. Chip.

[120] Wong C.H., Siah K.W., Lo A.W. (2019). Estimation of Clinical Trial Success Rates and Related Parameters. Biostat. Oxf. Engl..

[121] Wernitznig D., Meier-Menches M.S., Cseh K., Theiner S., Wenisch D., Schweikert A., Jakupec A.M., Koellensperger G., Wernitznig A., Sommergruber W. (2020). Plecstatin-1 Induces an Immunogenic Cell Death Signature in Colorectal Tumour Spheroids. Metallomics.

[122] Etminan N., Peters C., Lakbir D., Bünemann E., Börger V., Sabel M.C., Hänggi D., Steiger H.-J., Stummer W., Sorg R.V. (2011). Heat-Shock Protein 70-Dependent Dendritic Cell Activation by 5-Aminolevulinic Acid-Mediated Photodynamic Treatment of Human Glioblastoma Spheroids in Vitro. Br. J. Cancer.

[123] Silva P.D., Bano S., Pogue B.W., Wang K.K., Maytin E.V., Hasan T. (2021). Photodynamic Priming with Triple-Receptor Targeted Nanoconjugates That Trigger T Cell-Mediated Immune Responses in a 3D in Vitro Heterocellular Model of Pancreatic Cancer. Nanophotonics.

[124] Sebrell T.A., Hashimi M., Sidar B., Wilkinson R.A., Kirpotina L., Quinn M.T., Malkoç Z., Taylor P.J., Wilking J.N., Bimczok D. (2019). A Novel Gastric Spheroid Co-Culture Model Reveals Chemokine-Dependent Recruitment of Human Dendritic Cells to the Gastric Epithelium. Cell. Mol. Gastroenterol. Hepatol..

[125] Nyga A., Neves J., Stamati K., Loizidou M., Emberton M., Cheema U. (2016). The next Level of 3D Tumour Models: Immunocompetence. Drug Discov. Today.

[126] Weydert Z., Lal-Nag M., Mathews-Greiner L., Thiel C., Cordes H., Küpfer L., Guye P., Kelm J.M., Ferrer M. (2020). A 3D Heterotypic Multicellular Tumor Spheroid Assay Platform to Discriminate Drug Effects on Stroma versus Cancer Cells. SLAS Discov..

[127] Grönholm M., Feodoroff M., Antignani G., Martins B., Hamdan F., Cerullo V. (2021). Patient-Derived Organoids for Precision Cancer Immunotherapy. Cancer Res..

[128] Biselli E., Agliari E., Barra A., Bertani F.R., Gerardino A., De Ninno A., Mencattini A., Di Giuseppe D., Mattei F., Schiavoni G. (2017). Organs on Chip Approach: A Tool to Evaluate Cancer -Immune Cells Interactions. Sci. Rep..

[129] Tumor and Lymph Node on Chip for Cancer Studies | Tumor-LN-OC Project | Fact Sheet | H2020. https://cordis.europa.eu/project/id/953234.

[130] Galluzzi L., Vitale I., Warren S., Adjemian S., Agostinis P., Martinez A.B., Chan T.A., Coukos G., Demaria S., Deutsch E. (2020). Consensus Guidelines for the Definition, Detection and Interpretation of Immunogenic Cell Death. J. Immunother. Cancer.

[131] Aaes T.L., Verschuere H., Kaczmarek A., Heyndrickx L., Wiernicki B., Delrue I., De Craene B., Taminau J., Delvaeye T., Bertrand M.J.M. (2020). Immunodominant AH1 Antigen-Deficient Necroptotic, but Not Apoptotic, Murine Cancer Cells Induce Antitumor Protection. J. Immunol..

